# Managing burn victims of suicide bombing attacks: outcomes, lessons learnt, and changes made from three attacks in Indonesia

**DOI:** 10.1186/cc5681

**Published:** 2007-02-02

**Authors:** Harvey Chim, Woon Si Yew, Colin Song

**Affiliations:** 1 Department of Plastic Surgery and Burns, Singapore General Hospital, Block 4, Level 6, Outram Road, Singapore 169608, Singapore; 2 Department of Anaesthesia and Surgical Intensive Care, Singapore General Hospital, Block 5, Level 2, Outram Road, Singapore 169608, Singapore

## Abstract

**Introduction:**

Terror attacks in Southeast Asia were almost nonexistent until the 2002 Bali bomb blast, considered the deadliest attack in Indonesian history. Further attacks in 2003 (Jakarta), 2004 (Jakarta), and 2005 (Bali) have turned terrorist attacks into an ever-present reality.

**Methods:**

The authors reviewed medical charts of victims evacuated to the Singapore General Hospital (SGH) Burns Centre during three suicide attacks involving Bali (2002 and 2005) and the Jakarta Marriott hotel (2003). Problems faced, lessons learnt, and costs incurred are discussed. A burns disaster plan drawing on lessons learnt from these attacks is presented.

**Results:**

Thirty-one patients were treated at the SGH Burns Centre in three attacks (2002 Bali attack [*n *= 15], 2003 Jakarta attack [*n *= 14], and 2005 Bali attack [*n *= 2]). For the 2002 Bali attack, median age was 29 years (range 20 to 50 years), median percentage of total burn surface area (TBSA) was 29% (range 5% to 55%), and median abbreviated burn severity index (ABSI) was 6 (range 3 to 10). Eight of 15 patients were admitted to the intensive care unit. For the 2003 Jakarta attack, median age was 35 years (range 24 to 56 years), median percentage of TBSA was 10% (range 2% to 46%), and median ABSI was 4 (range 3 to 9). A large number of patients had other injuries. Problems faced included manpower issues, lack of bed space, shortage of blood products, and lack of cadaver skin.

**Conclusion:**

The changing nature of terror attacks mandates continued vigilance and disaster preparedness. The multidimensional burns patient, complicated by other injuries, is likely to become increasingly common. A burns disaster plan with emphasis on effective command, control, and communication as well as organisation of health care personnel following a 'team concept' will do much to ensure that the sudden onset of a crisis situation at an unexpected time does not overwhelm hospital manpower and resources.

## Introduction

Urban terrorism has been called the scourge of our times [1]. Indeed, the number and scale of terrorist attacks occurring in the past few years have been unprecedented, with devastating consequences and massive loss of life. The increasing prevalence of suicide bombing attacks, striking at unexpected times and places and oftentimes causing multidimensional injuries with components of penetrating trauma, blast injury, and burns [2], has made treating victims of these attacks a difficult and pressing concern. In addition, because victims of suicide bombing attacks are more severely injured compared with other trauma victims [3], with a large proportion requiring intensive care, hospital preparedness and formal protocols for dealing with mass casualty incidents (MCIs) are paramount.

In Southeast Asia, terrorist attacks were almost nonexistent until the 2002 bombing at Kuta Beach on the island of Bali. After this attack, considered the deadliest act of terrorism in Indonesian history, further attacks targeting the Jakarta Marriott hotel (Indonesia) in 2003 and the Australian embassy in Jakarta in 2004 and further Bali bombings in 2005 have turned terrorist attacks into an ever-present reality. Although those responsible for the attacks have been arrested and charged (the Jemaah Islamiah, an organisation allegedly affiliated with al-Qaeda, was held liable for the attacks), the victims and relatives involved on those fateful days will forever bear the scars of terrorism.

In three of these attacks, the Singapore General Hospital (SGH) Burns Centre served as a receiving facility for some of the most severely burned victims in the immediate aftermath of the blasts. SGH is a level I trauma centre, and the SGH Burns Centre, the only dedicated burn facility serving Singapore, receives 93% of total burns cases in Singapore, a city-state with a population of 4.18 million [4]. In addition, the SGH Burns Centre routinely receives severely burned patients throughout Southeast Asia requiring specialised burn care.

This report describes the characteristics of patients received in the aftermath of the 2002 and 2005 Bali bombings as well as the 2003 Jakarta Marriott hotel bombing, along with problems faced, the manner of response, lessons learnt, and costs incurred. In addition, a disaster plan for management of future terrorist incidents in Southeast Asia involving large numbers of burn victims is presented, drawn up from the experience of these three devastating attacks.

## Materials and methods

### Terror bomb incidents

#### The 2002 Bali bombing

On 12 October 2002 at 11:05 p.m. at Kuta, a town in southern Bali, a suicide bomber triggered a device hidden in a backpack, causing an explosion to tear through Paddy's Bar. Fifteen seconds later, in front of the Sari Club, a much larger car bomb of close to 1,000 kg concealed in a white van was detonated by remote control. The blast left a one meter-deep crater, and the shock wave blew out windows throughout the town. Scores of victims were killed and many more suffered severe trauma and burns. A third bomb had been detonated in front of the American consulate in Bali shortly before, causing only slight injury to one person. When all bodies were accounted for, it was found that 202 people had lost their lives [5]. Two hundred and nine people were injured, 15 with severe burns, and were evacuated to our centre.

#### The 2003 Jakarta Marriott bombing

On 5 August 2003, near the lunch hour in Jakarta, a car bomb exploded in the driveway of the Marriott hotel, killing 12 people and injuring another 150 [6]. The force of the explosion shattered windows 30 floors up, and the attack left bodies lying among shattered debris and wrecked cars in the street. Although this attack was smaller in scale than the preceding Bali blast, the psychological effect on the Indonesian people was no less marked, with terrorists striking with impunity in the heart of the capital city. Fourteen burn patients were evacuated to our centre.

#### The 2005 Bali bombing

On 1 October 2005 at 6:50 p.m. in Bali, two explosions caused by suicide bombers ripped through a Jimbaran Beach food court, and a third bomber struck at 7 p.m. in the main square of central Kuta. Unlike in previous attacks, many of the casualties sustained shrapnel injuries as well as injuries due to ball bearings, suggesting a different modus operandi for the bombers. The final death toll was 20, and another 129 were injured [7]. Most of the injured were sent to Bali's Sanglah General Hospital and treated largely for injuries caused by broken glass. Many of the casualties were foreign nationals. The two most severely injured victims, a father and daughter, were evacuated by air on 2 and 3 October to SGH for further management.

### SGH Burns Centre

The SGH Burns Centre is less than two hours by air from much of Indonesia and is located 1,050 miles from Denpasar, Bali, and 555 miles from Jakarta and therefore was ideally placed to receive casualties after these attacks. It is a 29-bed facility divided into a 4-bed intensive care unit (ICU), 6-bed high-dependency unit, and 19-bed general ward. After the 2002 Bali attack, the facility was renovated and the ICU is now able to nurse eight patients in a crisis situation as each of the cubicles is double-spaced (with patients housed as such only in a crisis with insufficient bed space). The mean annual admission to the Burns Centre is 288 patients [4]. The mean number of ICU admissions was 9 (3% of total admissions) (range 8 to 10) between 2003 and 2005. However, 16 patients were admitted in 2002 in the wake of the 2002 Bali attack. Patients were evacuated by air to our centre by the International SOS (a non-for-profit first-aid organisation) after initial stabilisation at Indonesian hospitals. Prior to evacuation, the International SOS corresponded with staff at the Burns Centre to ensure that the most severely injured victims were evacuated first. Upon arrival, patients were admitted directly to the Burns Centre for further management.

### Data collection

Data on patients were obtained from a retrospective review of medical records. Information on demographic data, injuries sustained, complications, surgeries, and outcome was obtained. Information on costs incurred in the wake of the terrorist attacks was obtained from records that were kept by the finance office of SGH and based on hospital bills incurred by individual patients. Data on cadaver skin obtained and skin-banking protocols were obtained from the skin bank at the SGH Burns Centre. Information regarding the events surrounding previous terrorist attacks was obtained from the news media.

## Results

### Characteristics of burn patients treated

From October 2002 to October 2005, the SGH Burns Centre was involved in the management of 31 patients evacuated from three separate suicide bombing attacks in Indonesia. Table 1 presents the characteristics of patients evacuated to our centre. For the 12 October 2002 Bali bomb blast, 15 patients were evacuated to Singapore on 14 October after stabilisation and triage at local hospitals. Many of these patients were severely burned (median percentage of total burn surface area [TBSA] of 29%) and eight patients were admitted to the ICU. Patients admitted to the ICU either had inhalational burns or required intubation due to severe burns. All patients with suspected inhalational injury had a diagnostic bronchoscopy. One patient died of multiorgan failure with septicaemia and pneumonia following a protracted ICU stay (24 days after admission), but the others survived. Eleven patients required surgery; a total of 36 burn surgeries and 3 non-burn surgeries were performed.

**Table 1:** Characteristics of burn patients treated in the three attacks

	All attacks	Bali 2002	Jakarta 2003	Bali 2005
Number of victims	31	15	14	2
Age in years^a^	32 (13–56)	29 (20–50)	35 (24–56)	28 (13–43)
Gender (male/female)	17:14	6:9	10:4	1:1
Percentage of TBSA^a^	15 (2–55)	29 (5–55)	10 (2–46)	11.5 (7–16)
ABSI^a^	5 (2–10)	6 (3–10)	4 (3–9)	3.5 (2–5)
Inhalational injury^b^	2 (6%)	0 (0%)	2 (14%)	0 (0%)
Admitted to the ICU^b^	10 (32%)	8 (53%)	2 (14%)	0 (0%)
Number of burn surgeries^a^	2 (0–10)	2 (0–10)	2 (0–6)	1 (1–1)
Length of hospital stay in days^a^	11 (2–58)	6 (2–42)	16.5 (7–58)	10 (9–11)
Mortality^b^	1 (3%)	1 (7%)	0 (0%)	0 (0%)

^a^Data presented as median (range). ^b^Data presented as number (percentage of total). ABSI, abbreviated burn severity index; TBSA, total burn surface area.

In contrast, for the 5 August 2003 Jakarta Marriott hotel bombing, patients evacuated to our centre were less severely burned (median percentage of TBSA of 10%) and only two patients were admitted to the ICU. All patients survived. Thirteen patients required surgery; 29 burn surgeries and 7 non-burn surgeries were performed. Patients were evacuated to Singapore in waves from 6 August to 9 August, and the two most severely injured requiring ICU care arrived first. This likely reflects the smaller scale of the 2003 Jakarta bombing as opposed to the three bombs detonated in the 2002 Bali bombing.

Interestingly, all the patients from the 2002 Bali bombing treated at the SGH Burns Centre were non-Indonesian (comprising a mix of American, British, Swiss, French, Irish, Canadian, Singaporean, and Japanese nationals) and of relatively young age (median 29 years). The terrorists targeted crowded areas frequented by tourists, and this likely explains why many foreign nationals sustained severe burn injuries. At the request of patients and national authorities, six of these patients were evacuated to their home countries after a two to eight day hospitalisation in Singapore (where essential surgery and resuscitation were performed). In contrast, for the 2003 Jakarta bombing, all the patients seen were either Indonesian or Singaporean, perhaps due to lesser numbers of foreign tourists visiting Indonesia.

Table 2 shows characteristics of patients admitted to the ICU. Although a direct comparison cannot be made with other terror attacks (because select patients were evacuated to our centre), it is useful to make a comparison. In the Israeli experience [8], 55% of patients with burns or penetrating injuries required ICU care and median length of stay was 4 days. Our experience was similar for the 2002 Bali bomb blast; 53% of patients were admitted to the ICU and median length of ICU stay was 4.5 days. The median length of stay for ICU patients was the same (at 4.5 days) for patients admitted to Gregario Maranon University General Hospital after the Madrid (Spain) train attack in March 2004 [9]. This is in accordance with previous studies showing that terror victims stayed in the ICU considerably longer than other ICU patients [3]. The severity of burn injury for patients admitted to the ICU in the 2002 Bali attack (average percentage of TBSA of 39%) was similar to that observed for burn patients requiring critical care in the 9/11 Pentagon attack [10] (average percentage of TBSA of 34%). Of the two patients admitted to the ICU after the 2003 Jakarta attack, one had a protracted ICU stay with prolonged ventilation as he developed acute respiratory distress syndrome with pneumonia and septicaemia.

**Table 2:** Characteristics of burn patients admitted to the ICU

	All attacks	Bali 2002	Jakarta 2003
Number	10	8	2
Age in years^a^	29.5 (23–56)	28.5 (23–35)	45.5 (35–56)
Gender (male/female)	3:7	1:7	2:0
Percentage of TBSA^a^	37.5 (23–55)	37.5 (23–55)	39.5 (33–46)
ABSI^a^	8 (6–10)	7.5 (6–10)	8.5 (8–9)
Ventilator days^a^	3.5 (1–40)	3 (1–18)	24 (8–40)
Length of ICU stay in days^a^	6 (2–40)	4.5 (2–24)	24.5 (9–40)

^a^Data presented as median (range). ABSI, abbreviated burn severity index; ICU, intensive care unit; TBSA, total burn surface area.

A large number of the patients seen at the SGH Burns Centre had other injuries as shown in Table 3. The most common concomitant injury seen was ear barotrauma. Eight patients (53%) from the 2002 Bali attack and both from the 2005 Bali attack (100%) had ear barotrauma. In contrast, only one patient from the 2003 Jakarta attack (7%) had barotrauma. This could be explained by the different settings of the attacks. In the Bali attacks, victims were directly exposed to the full force of the blasts. However, in the Jakarta Marriott attack, which involved the detonation of a car bomb in the driveway of the hotel, victims were likely shielded from the blasts by the hotel walls. Of patients presenting with ear barotrauma, one from the 2002 Bali attack (7%) had other primary blast injuries as well, including a pneumothorax of the left lung. The most severely injured victim of the 2005 Bali bombing, a 43-year-old man who was evacuated to Singapore, had ear barotraumas and also sustained secondary blast injuries, including a ruptured spleen and fractures as well as injuries from multiple ball bearings lodged in his thorax, abdomen, and spine, causing Brown-Séquard syndrome.

**Table 3:** Number of patients with other injuries admitted to the Singapore General Hospital Burns Centre

	All attacks	Bali 2002	Jakarta 2003	Bali 2005
Number	31	15	14	2
Ear barotrauma	11 (35%)	8 (53%)	1 (7%)	2 (100%)
Fractures/Dislocations	7 (23%)	4 (27%)	2 (14%)	1 (50%)
Pneumothorax	3 (10%)	2 (13%)	1 (7%)	0 (0%)
Ruptured spleen	1 (3%)	0 (0%)	0 (0%)	1 (50%)
Neurological injury	2 (6%)	0 (0%)	1 (7%)	1 (50%)
PTSD	3 (10%)	2 (13%)	0 (0%)	1 (50%)
Shrapnel wounds	4 (13%)	1 (7%)	2 (14%)	1 (50%)
Lacerations	3 (10%)	2 (13%)	7 (50%)	1 (50%)
Tendon injuries	3 (10%)	0 (0%)	3 (21%)	0 (0%)

All data are presented as number (percentage of total). PTSD, post-traumatic stress disorder.

### Problems encountered and solutions used

Throughout the three terrorist attacks, the SGH Burns Centre continued to function normally, admitting burn patients from Singapore and abroad. This was unavoidable given that we are the only regional burns centre in this part of Southeast Asia. The main problems faced were those of manpower, lack of bed space, shortage of blood products, and lack of cadaver skin. With the sudden influx of 15 patients in one day after the 2002 Bali bomb blast, the usual staff complement of the Burns Centre was insufficient to manage the situation. As a result, off-duty staff were recalled, additional critical care trained nurses were recruited from the surgical and medical ICUs, and surgical residents who had previously done a burns rotation were seconded to assist in managing patients in the week following the incident. The nine plastic surgeons in the unit with teams of surgical residents worked 12-hour shifts in the days following the attack, operating on the patients. Additional operating theatres were allocated for use in the management of victims of the Bali bomb blast. The estimated number of cancelled elective surgeries, particularly those requiring ICU care, was 20 to 30.

Of the eight patients requiring ICU care after the 2002 Bali attack, four were housed in the surgical ICU, managed with the aid of additional surgical intensivists recruited during this period. Patients judged fit enough for step-down care were discharged from the surgical ICU prior to the arrival of the first patients from Bali. The 2003 Jakarta and 2005 Bali attacks did not pose such a major manpower and resource problem; this was due to a smaller number of patients requiring critical care and surgery as well as better disaster preparedness resulting from the experience of managing patients from the 2002 attack.

In our burns centre, we practice early massive excision of burns and temporary coverage by skin substitutes followed by definitive wound closure with autologous skin grafts in staged surgeries. As a result, a significant problem encountered after the 2002 Bali bombing was an acute shortage of blood products following the many surgeries performed for victims of that attack. Fortunately, the National Blood Bank was able to obtain more blood at short notice through blood donation drives and recalling regular blood donors during this period. However, to conserve blood products, many elective surgeries were also cancelled in the immediate aftermath of the attack.

The lack of cadaver skin was another major issue after the 2002 Bali bomb blast. Prior to this attack, no contingency had ever called for the massive amounts of cadaver skin required. On 14 October 2002, when the patients arrived from Bali, there was 9,100 cm^2 ^of cadaver skin in our skin bank. By 18 October, only 5,450 cm^2 ^was left. Fortunately, a prior request for substitute skin had been made to the University of Texas Southwestern Medical Center at Galveston (TX, USA), which responded by sending 22,968 cm^2 ^of skin to Singapore. The shipment arrived on 18 October 2002, forestalling the anticipated shortage of cadaver skin. Better prepared for the 2003 Jakarta and 2005 Bali attacks, the SGH Burns Centre did not face any further shortages of skin substitutes.

### Cost of the terrorist attacks

The cost of these consecutive suicide bombing attacks, at regular intervals in Indonesia, to the victims and families involved cannot be quantified. Numerous people lost their lives, and many more were injured. In addition, the previous absence of terror attacks was replaced by an almost annual bomb blast in Indonesia, changing the region forever. The psychological effect has been no less, with a pervading sense of danger among the local populace and marked decrease in tourism to the region seen after these attacks.

In terms of costs incurred by patients during their stay, the 2002 Bali bombing cost SGD $765,702 (USD $450,412). The 2003 Jakarta bombing cost SGD $603,008 (USD $354,710), and the cost of the 2005 Bali bombing was SGD $38,535 (USD $22,667).

## Discussion

The number of patients suffering burns as a result of the 2002 Bali bombing was extremely high; 15 patients were treated at the SGH Burns Centre, and a further 48 were evacuated from the Royal Darwin Hospital (Tiwi, NT, Australia) to Australian burns centres [11]. We will never know of the many more patients who were not evacuated from Bali. In contrast, only 18 burn patients were transferred to the Cornell Burn Center (New York, NY, USA) [12] after the September 11 attacks in New York and 9 patients were admitted to the Washington Hospital Center Burn Center (Washington, DC, USA) after the 9/11 attack on the Pentagon [10]. The scale of the 2002 Bali attack may best be appreciated by the fact that in Israel, over a period of two years, only 91 burn patients (out of a total of 623 victims injured by terror-related explosions) [8] in multiple terror attacks were hospitalised. The 2003 Jakarta Marriott bombing was of smaller scale (only 14 patients were evacuated) but was still significant in relation to other terror attacks of these times.

Burns centres are never the first responders in terrorist attacks. However, they almost invariably play a pivotal role in the subsequent management of burn patients. A formalised protocol for MCIs and limited MCIs is therefore essential to ensure proper workflow during a crisis situation as well as the ability to cope with a massive surge in patients transferred at short notice. The experience of previous terrorist attacks would suggest that the number of burn patients to be expected is significant. In Israel, of patients hospitalised after injury by terror-related explosions, 15% suffered burns [8]. After 9/11 in New York City, 14% of patients admitted to Bellevue Hospital and New York University Downtown Hospital were burn patients [12]. Similar figures were reported at St. Vincent's Hospital, where 19% of those hospitalised were burn patients [13].

Due to Singapore's location at the crossroads of air and sea traffic, as well as the presence of a petrochemical industry and high-density urban sprawl, burns preparedness has always been a priority. The SGH Burns Centre Burns Disaster Plan was conceptualised and designed in light of lessons learnt after the recent terror attacks in Indonesia. It emphasises effective command, control, and communication as well as a 'team concept' in which medical and nursing personnel are organised into teams for better management of burns patients. Yearly drills ensure that staff are kept up to date on processes and procedures. Future validation of the disaster plan is planned. The major problems faced during the Indonesian terror attacks – such as manpower issues, lack of bed space, and resource shortage – were analysed and solutions proposed. Cross-training of personnel to provide additional manpower in a crisis situation and provision for opening of additional ICUs and wards were instituted. In addition, a directive from the Ministry of Health, Singapore, stipulated requirements for a minimum supply of cadaver skin to be banked at the Burns Centre at all times.

**Figure 1:**
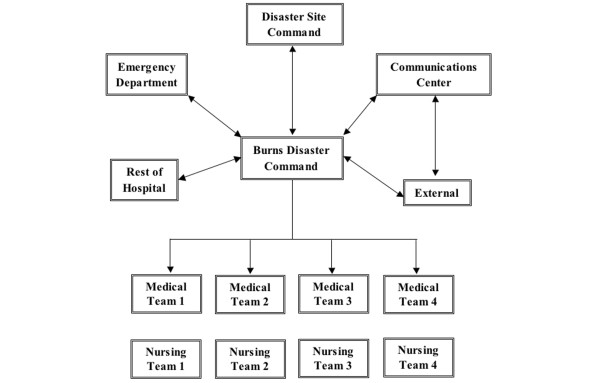
Organisation of personnel in the Singapore General Hospital Burns Centre during a crisis situation. Emphasis is placed on effective command, control, and communication and a 'team concept.'

In a crisis situation, a Burns Disaster Command is formed with the director of the Burns Centre serving as the director of operations. He is assisted by a team comprised of senior nursing staff and administrative and communications officers. The Burns Disaster Command is housed in a specialised room serving as an operations centre and provides command and control of all Burns Centre personnel (Figure 1). Medical staff are organised into teams comprised of one attending physician (team leader), one senior resident, one junior resident, and one staff nurse attached directly to the medical team. Nursing staff are also organised into individual teams under the direction of each medical team. Each team, when activated, is responsible for a specific task in the initial phase of the crisis (for example, resuscitation, performing investigations, or clerking patients). Subsequently, when the situation has stabilised somewhat, each team takes on responsibility for the care of a specific group of patients and takes turns admitting patients to prevent the same group of doctors or nurses from being overwhelmed by a sudden surge of casualties. When activated, the team leader is stationed at the emergency department (ED) to triage patients while the rest of the team is stationed in the ward.

Contingency plans also allow for the opening of additional temporary ICUs during an MCI in areas such as the operating theatre recovery rooms, endoscopy suite, and day surgery suite. Intensivists are organised into teams comprised of four attending physicians and four residents, with one team covering each ICU.

A dedicated briefing and communications room for press conferences and communication with family members is manned by a communications officer, who has easy access to social workers, psychologists, nurses, and doctors. To ensure that effective channels of communication are maintained, dedicated phone lines are maintained between the operations centre, communications room, ED, disaster site command, and the rest of the hospital. The operations centre serves as the nerve centre of the communications network. A plan for activation and recall of medical and nursing staff is in place with varying levels of activation, and the level of activation of personnel is decided by the director of the Burns Centre. The role of local authorities in a mass casualty situation is also vital for preserving lines of communication and transport. Civil defence and emergency services contingency plans are in place to prepare for such a situation.

**Figure 2:**
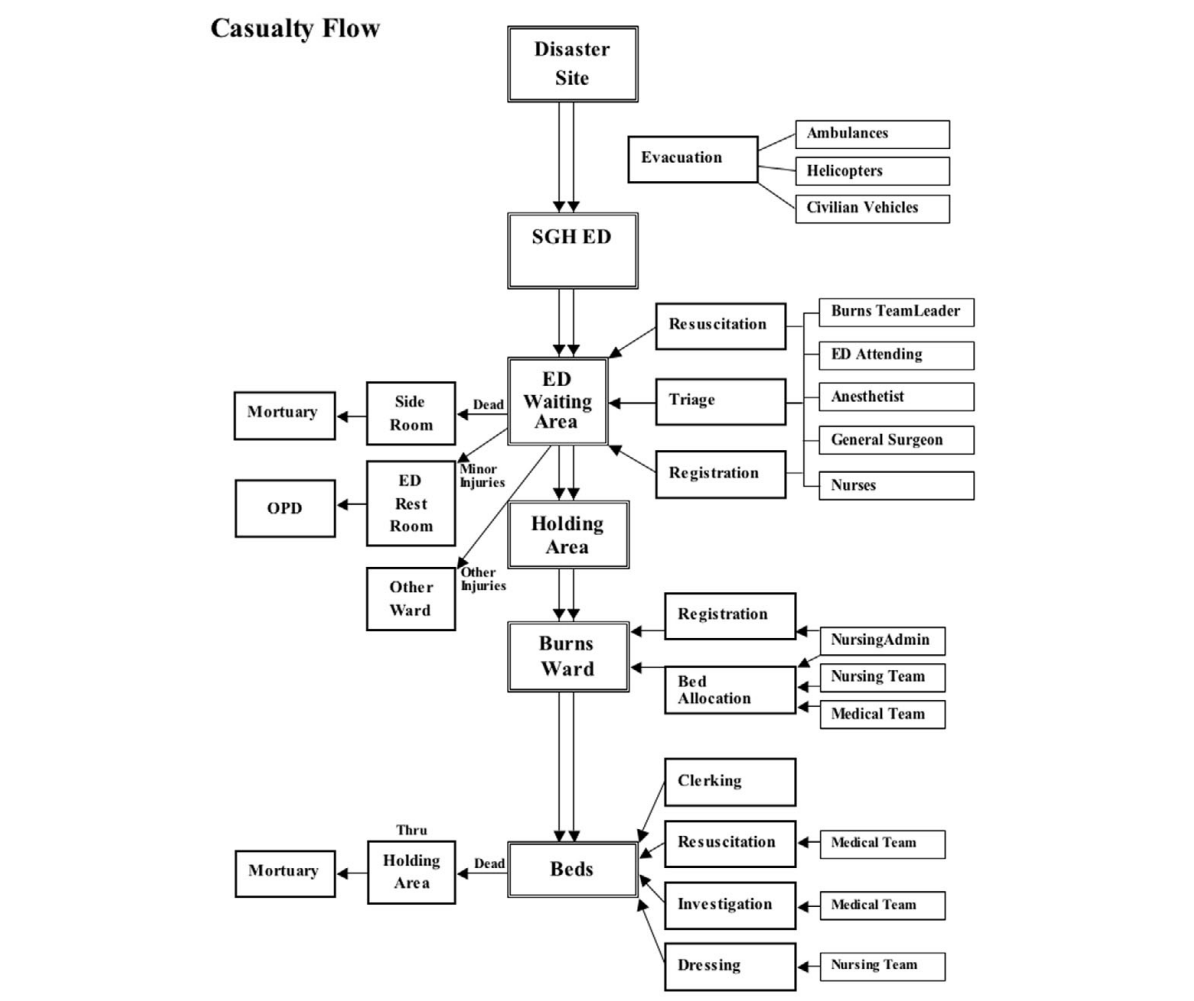
Unidirectional flow of casualties is essential to ensure that health care personnel are able to cope with the flood of patients and that adequate care is provided to all victims in a mass casualty situation. ED, emergency department; OPD, outpatient department; SGH, Singapore General Hospital.

Unidirectional flow of casualties is a priority and is formalised into a protocol for reference of medical and nursing personnel (Figure 2). In addition, admissions are staggered to prevent staff in the burns ward from being overwhelmed, and the observation ward in the ED serves as a holding area for patients. Debriefings are conducted regularly, and all medical and nursing teams meet regularly and at daily intervals when the situation has stabilised to prioritise patients for surgery and discuss allocation of hospital resources and other pressing issues. Although the immediate aftermath of an MCI will prove a strain to hospital resources, a formalised disaster plan will do much in the midst of a crisis to ensure that good care is provided to all victims and that medical and nursing staff are well looked after. At the national level, in a further effort to ensure that sufficiently trained staff are available in the event of a mass casualty situation, cross-training of nursing staff was instituted; surgical and critical care nurses in particular are required to rotate through the Burns Centre. A burns course targeted at surgical residents was also instituted to ensure that all surgical residents would be equipped to manage burn patients if called upon.

Despite all possible preparative measures and a formalised burns disaster plan to forestall chaos, in a true mass casualty scenario, manpower and hospital resources may still be insufficient to cope with the situation. The concept of 'minimal acceptable care' in terror attacks, in which effort is concentrated on a maximal number of salvageable patients [8,14,15], has been proposed to optimise evacuation preferences and guide triage as well as determine to which patients critical hospital resources are allocated. A modification of this concept may be applicable in burns patients. Because most burns centres now practice early massive burns excision with immediate cover by autologous skin and skin substitutes within 72 hours of the initial burn injury [4,16], the strain on staff and resources such as cadaver skin in a true MCI may force burn surgeons to select patients to be operated on. Those with the maximal chance of survival – patients who are moderate in the severity of their burns, who require early burns excision to optimise recovery, and who are not so severely burned that they are likely to develop other complications – would be the natural candidates for surgical priority. In the event of a severe shortage of cadaver skin, temporary skin substitutes such as Silon-TSR (STAT Pharmaceuticals, Inc., Santee, CA, USA), a semi-occlusive non-adherent dressing, or Biobrane (Mylan Laboratories Inc., Canonsburg, PA, USA) can be used to cover the burn wound and promote epithelisation until autologous skin from the patient can be harvested.

There appears to be a clear difference between normal burn patients and victims of terror attacks requiring admission to a burns centre. In our experience, a large proportion of victims of terror attacks have had multidimensional injury with other injuries besides burns. Similarly, in the Israeli experience, 68% of patients with burn injuries had penetrating and blunt injuries [8]. This is exemplified by the 43-year-old man previously described, injured in the 2005 Bali bombing, who also sustained spinal cord injury, fractures, and penetrating injuries to the thorax and abdomen caused by multiple ball bearings. If the changing nature of suicide bombing attacks in Indonesia is an indication of tidings to come, with increasing use of ball bearings and heavy shrapnel in bombs, future victims of terror attacks with burns are increasingly likely to present with multiple injuries besides the burn injury. The multidimensional burns patient, particularly one requiring critical care, presents a particular therapeutic challenge. He has an increased operative risk due to multiple injuries but requires surgery more than others to forestall problems related to delayed burns excision. To compound the problem, after burns excision, he is at increased risk of metabolic derangements and multiorgan failure due to other injuries and therefore will likely require prolonged ICU support. Burns centres should be prepared for managing such difficult and unstable patients as part of disaster preparedness.

For patients with burn injuries sustained in bomb blasts, it must be appreciated that the blast wave caused by heavy explosives results in an additional element of soft tissue destruction. This is especially true for those victims closest to the blasts. These wounds cannot be treated primarily as burn wounds but require repeated reassessment in the subsequent 48 hours for progression to deeper tissue destruction. Therein lies the problem for MCIs in which facilities and staff are overwhelmed and the resultant level of care is therefore suboptimal.

## Conclusion

The changing nature of terror attacks mandates continuing vigilance and disaster preparedness. The multidimensional burns patient, complicated by other injuries, is a particular challenge to manage but is likely to become increasingly common. A structured burns disaster plan with emphasis on control, command, and communication will do much to ensure that the sudden onset of a crisis situation at an unexpected time does not overwhelm hospital manpower and resources. In extreme circumstances, 'minimal acceptable care' with the selective treatment of burn patients to conserve hospital resources and maximise manpower may offer an alternative to an overwhelmed health care system.

## Key messages

• A burns disaster plan with emphasis on effective command, control, and communication as well as organisation of health care personnel following a 'team concept' will do much to ensure that the sudden onset of a crisis situation at an unexpected time does not overwhelm hospital manpower and resources.

• The changing nature of terror attacks mandates continued vigilance and disaster preparedness.

• In a mass casualty situation after a terrorist attack, 'minimal acceptable care' with the selective treatment of burn patients to conserve hospital resources and maximise manpower may offer an alternative to an overwhelmed health care system.

• After a terror attack, the multidimensional burns patient, complicated by other injuries, is likely to become increasingly common, and hospitals should prepare to treat this kind of patient.

## Abbreviations

ED = emergency department; ICU = intensive care unit; MCI = mass casualty incident; SGH = Singapore General Hospital; TBSA = total burn surface area.

## Competing interests

The authors declare that they have no competing interests.

## Authors' contributions

HC conceived the study, carried out the research, and wrote the manuscript. WSY and CS participated in care of patients and helped to draft the manuscript. All authors read and approved the final manuscript.
